# Solomonseal Polysaccharide and Sulfated *Codonopsis pilosula* Polysaccharide Synergistically Resist Newcastle Disease Virus

**DOI:** 10.1371/journal.pone.0117916

**Published:** 2015-02-18

**Authors:** Cui Liu, Jin Chen, Entao Li, Qiang Fan, Deyun Wang, Cunshuai Zhang, Peng Li, Xiuping Li, Xingying Chen, Shulei Qiu, Zhenzhen Gao, Hongquan Li, Yuanliang Hu

**Affiliations:** 1 Institute of Traditional Chinese Veterinary Medicine, College of Veterinary Medicine, Nanjing Agricultural University, Nanjing, 210095, PR China; 2 National Research Center of Veterinary Biological Engineering and Technology, Jiangsu Academy of Agricultural Sciences, Nanjing, 210014, PR China; 3 China Institute of Veterinary Drug Control, Beijing, 100081, PR China; 4 College of Animal Science and Veterinary Medicine, Shanxi Agricultural University, Taigu, Shanxi, 030801, PR China; Fondazione IRCCS Policlinico San Matteo, ITALY

## Abstract

Five combinations of three ratios (PS_9_-sPS_1_, PS_7_-sPS_3_ and PS_6_-sPS_4_) were prepared with polysaccharide (PS) and sulfated polysaccharide (sPS). The antiviral activities of these compounds were subsequently compared in vitro using the MTT assay, observation of the virus structure and immunofluorescence. The results demonstrated that SP_9_-sCP_1_, CP_7_-sCA_3_, EP_7_-sAP_3_, CA_9_-sEP_1_ and EP_7_-sCA_3_ presented higher activities, and SP_9_-sCP_1_ displayed the highest virus inhibition rate and clearly killed the virus and inhibited viral antigen expression. In an in vivo test, 28-day-old chickens were challenged with Newcastle disease virus (NDV) and were administered the five drug combinations. On day 14 after the challenge, the morbidity, mortality and cure rate in each group were calculated. The results indicated that SP_9_-sCP_1_ presented the lowest morbidity and mortality and the highest cure rate. These results indicate that Solomonseal polysaccharide and sulfated *Codonopsis pilosula* polysaccharide synergistically resist NDV. Moreover, SP_9_-sCP_1_ had the highest efficacy and may be used as a new antiviral drug.

## Introduction

Viral infectious diseases of animals are of worldwide concern because they cause great economic losses in the domestic animal and poultry industries. Vaccination is the most effective preventive measure, but several infectious diseases occur despite intensive vaccination [1]. After an infectious viral disease outbreak, there are no effective cure methods. Several cytokines may have a curative effect, but there remain obstacles to their use, such as the cost for large-scale production, effective delivery, and retention of protein stability and bioactivity in vivo [2]. Therefore, it is necessary to identify and develop new types of antiviral drugs with high efficacy and low toxicity.

Many studies have demonstrated that Chinese herbal medicines and ingredients (CHMIs), such as polysaccharides, flavones and saponin possess antiviral activity [3], and several CHMIs were more potent in combination than alone [4]. Sulfated polysaccharides, including natural sulfated polysaccharides extracted from plants or derivatives synthesized from natural neutral polysaccharides, have greater biological activity, particularly antiviral activity [5–7]. Because natural sulfate polysaccharides are rare, the sulfation modification is widely used to improve the biological activities of the polysaccharides and obtain a greater amount of sulfated polysaccharides. Our previous studies showed that the sulfation modification significantly improved the antiviral activity of several polysaccharides, such as astragalus polysaccharide, epimedium polysaccharide, lentinan, tremella polysaccharide and *Auricularia auricula* polysaccharide [8–12].

Further studies showed that compound sulfated polysaccharides exhibited better efficacy. However, the combination of all synthesized sulfated polysaccharides was unsuitable for veterinary clinical application because of the higher cost and reduced safety. When a sulfated polysaccharide (sPS) in the drug combination was partially substituted with the corresponding polysaccharide (PS), the efficacy and safety of the combination (PS-sPS) were not lowered, whereas the cost was greatly decreased. The following five combinations of compounds were selected: *Codonopsis pilosula* polysaccharide plus sulfated Chinese angelica polysaccharide (CP-sCA), epimedium polysaccharide plus sulfated astragalus polysaccharide (EP-sAP), epimedium polysaccharide plus sulfated Chinese angelica polysaccharide (EP-sCA), Chinese angelica polysaccharide plus sulfated epimedium polysaccharide (CA-sEP), and solomonseal polysaccharide plus sulfated *Codonopsis pilosula* polysaccharide (SP-sCP).

In the present study, the five combinations described above were prepared at three ratios, 9:1 (PS_9_-sPS_1_), 7:3 (PS_7_-sPS_3_) and 6:4 (PS_6_-sPS_4_), according to their carbohydrate content. Their antiviral activities in vitro were compared by MTT assay, observation of the viral structure and immunofluorescence. The optimal ratios were then determined and their antiviral activities in vivo were validated with a challenge experiment using NDV in chickens. The objective of this study was to identify the combination of compounds with the strongest efficacy for the development of new antiviral drugs.

## Materials and Methods

### Preparation of polysaccharides and drug combinations

CP, EP, CA, and SP had carbohydrate contents of 87.4%, 71.23%, 91.7%, and 79.4%, respectively, and sCA, sAP, sEP and sCP had carbohydrate contents of 77.3%, 37.7%, 72.4%, and 63.2%, respectively, and a degree of substitution (DS) of 3.02, 1.545, 0.696, and 1.83 respectively. The Institute of Traditional Chinese Veterinary Medicine of Nanjing Agricultural University supplied these compounds.

**Fig 1:**
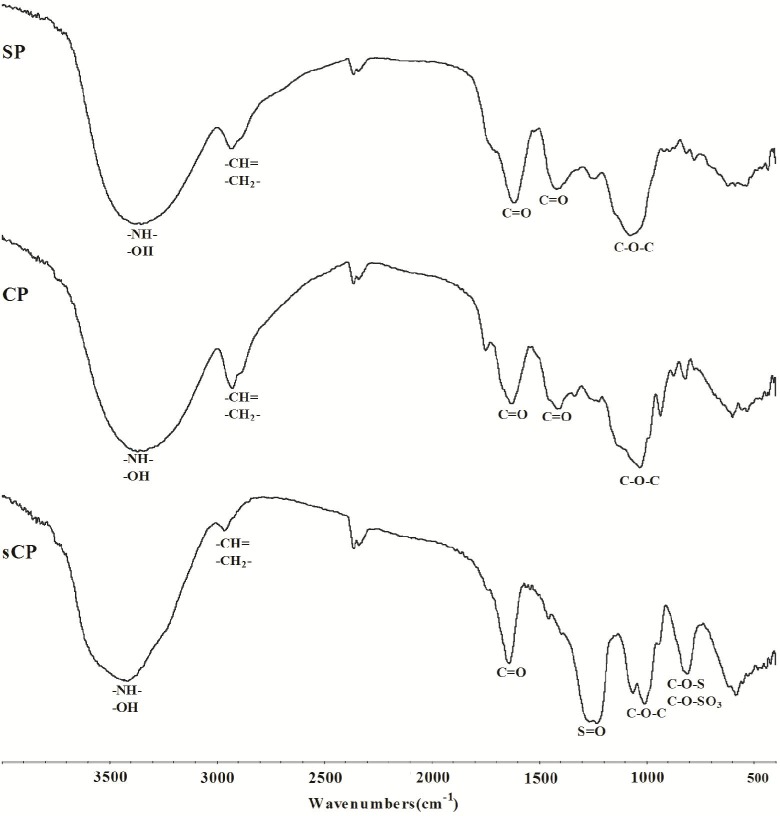
The FT-IR spectra of SP, CP and sCP.

The structures of these compounds were determined with Fourier transform-infrared spectroscopy [13]. The spectra of SP, CP and sCP (Fig. 1) contained two characteristic absorption bands for polysaccharide, with one in the region of 3500–3200 cm^-1^ corresponding to the -OH stretching vibration and the other in the region of 1600–1400 cm^-1^ that is characteristic of a carboxylic group. In the spectrum of sCP, a large S = O stretching vibration appeared at 1229.66 cm^-1^, and a symmetrical C-O-S stretching vibration appeared at 811.30 cm^-1^, which indicated that sCP was successfully in sulfated.

Five combinations, CP-sCA, EP-sAP, EP-sCA, CA-sEP and SP-sCP, at three ratios according to the carbohydrate content, PS_9_-sPS_1_, PS_7_-sPS_3_ and PS_6_-sPS_4_, for a total of 15 preparations were prepared. These were diluted to 1 mg·mL^-1^ (total content of polysaccharide) with deionized water, sterilized and stored at 4°C.

### Reagents

Eagle’s minimum essential medium (MEM) (Gibco, USA) supplemented with penicillin 100 IU·mL^-1^, streptomycin 100 IU·mL^-1^ and 5% fetal bovine serum was used as growth medium for culturing the cells, and medium with 2% fetal bovine serum was used as the maintenance medium (MM) for diluting the polysaccharides and maintaining the cells. The pH of the Hank’s solution was adjusted to 7.4 with 5.6% NaHCO_3_. Trypsin (Amresco-0858) was dissolved at a concentration of 0.25% with phosphate-buffered saline (PBS, pH 7.4). The 3-(4, 5-dimethyithiazol-2-yl)-2, 5- diphenyltetrazolium bromide (MTT, Amresco) was dissolved at 5 mg·mL-1 in PBS. These reagents were filtered through a 0.22 μm filter. MEM and MM were stored at 4°C. The trypsin solution was stored at −20°C, and the MTT solution was stored at 4°C in dark bottles. Dimethyl sulfoxide (DMSO, No. 30072418) was a product of the Chemical Agent Company of Chinese Medicine Groups, Nanjing.

Bovine serum albumin (BSA) was purchased from Aladdin Reagent Co., Ltd (No. Al16563) and diluted to 1% with PBS, which was used as blocking solution. Triton X-100 was purchased from Shanghai Yuanye Biotechnology Co., Ltd (No. 9002931) and dissolved at 0.3% with PBS. DAPI was purchased from the Beyotime Institute of Biotechnology (No. C1005). Anti-NDV ribonucleoprotein antibody [B1386M] and FITC-labeled goat anti-mouse IgG were purchased from Abcam. The other chemicals used were of analytical grade.

ND vaccine virus (La Sota strain IV, No.129101) was purchased from Nanjing Tian Bang Biotechnology Co., Ltd and was propagated in 9-day-old specific pathogen-free (SPF) chicken embryos. The TCID_50_ was 1×10^–6^ by Reed-Muench assay [14] after the allantoic fluid was concentrated by ultracentrifugation and dissolved in PBS. This preparation was used for the observation of virus structure and immunofluorescence. It was diluted 10^–4^ (100 TCID_50_) with 2% MEM for the MTT assay.

ND virus (F_48_E_9_ strain) was supplied by the China Institute of Veterinary Drug Control and was propagated in 9-day-old SPF chicken embryos. The LD_50_ was determined using 21-day-old White Roman chickens before the in vivo test.

### Determination of in vitro activity

#### MTT assay

All combinations were diluted into ten working concentrations (500–0.977 μg·mL^-1^) using a two-fold series with MM, and the maximal safe concentration for CEF were first measured by MTT assay [15,16]. The results showed that the maximal safe concentrations of all combinations ranged from 31.25–7.813 μg·mL^-1^. To compare identical concentrations of the drug combinations, the safe concentration was set as 7.813 μg·mL^-1^. The CEF were cultivated in a 5% CO_2_ incubator at 38.5 C. After the CEF grew into a monolayer after approximately 24 h, 100 μL per well ND vaccine virus solution was added. After incubation for 2 h, the virus solution was removed and the cells were washed twice with Hank’s solution. The two-fold serial dilutions ranging from 7.813 to 0.488 μg·mL^-1^ of the 15 combinations were then added to the plates in quadruplicate. The virus control (VC) group (virus only), cell control (CC) group (MM only) and blank control (BC) group (no cells) were also established. All plates were placed into a 5% CO_2_ incubator at 38.5°C. When the VC group showed a clear cytopathic effect (72 h), the cell *A*_570_ value was measured using the MTT assay. The highest virus inhibitory rate was calculated based on the following formula [17]: highest virus inhibitory rate = (the highest *Ā*_prescription+virus_−*Ā*_virus control_) */* (*Ā*_cell control_ −*Ā*_virus control_) × 100%.

#### Virus structure observation

Based on the results of the MTT assay, the effects of SP_9_-sCP_1_ on the NDV structure were observed by electron microscopy. The ND vaccine virus fluid (part of the control group) was mixed with SP_9_-sCP_1_ at 7.813 μg·mL^-1^ in an equal volume and incubated at 37°C (experimental group). At 30, 60 and 120 min after incubation, the virus solution (experimental and control groups) was dipped on 200 mesh var/carbon-coated copper grids, negatively stained with 2% phosphotungstic acid for 3–5 min and air dried. The virus was observed and photographed using transmission electron microscopy (Tecnai 12, Philips company, Holland) [18].

#### Immunofluorescence assay

Based on the results of the MTT assay, the effects of SP_9_-sCP_1_ on NDV antigen expression were observed by immunofluorescence assay. The CEF were cultivated on cover slips. After the CEF grew into a monolayer, 100 μL ND vaccine virus solution (multiplicity of infection [MOI] = 1.0) was added to the experimental group and virus control group for 2 h at 38.5°C. The virus solution was then removed from the experimental group and the cells were washed twice with Hank’s solution. Next, 100 μL SP_9_-sCP_1_ at 7.813 μg·mL^-1^ was added to the in virus control and cell control groups in 100 μL MM. After 48 h of infection, all cells were washed thrice with PBS, and fixed in 4% paraformaldehyde for 10 min at 4 C. Triton X-100 (0.3%) was added for 10 min to permeabilize the cells, 1% BSA was added for 1 h to block, and anti-NDV ribonucleoprotein antibody (1:300) was added for an overnight incubation in a wet chamber at 4°C, FITC-labeled goat anti-mouse IgG (1:100) was for 30 min in a wet chamber at 37°C. Finally, the cells were washed twice with PBS, stained with DAPI for 5 min at 4°C, washed with PBS and observed under a laser scanning confocal microscope (LSM 700, Zeiss, German). The fluorescence intensities of every group were quantitatively analyzed using ZEN lite 2011 software, the mean pixel intensities were calculated and expressed as pixel×10^2^/μm^2^ [19]. The higher value of pixel intensity, the stronger NDV antigen expression was.

### Determination of test in vivo

Based on the results of the in vitro test, five combinations, SP_9_-sCP_1_, CP_7_-sCA_3_, EP_7_-sAP_3_, CA_9_-sEP_1_ and EP_7_-sCA_3_, were selected for further study. Their curative effects were compared in chickens challenged with NDV.

#### Ethics statement

All animal experiments were treated in accordance with the guideline of Institutional Animal Care and Use Committee (IACUC), Nanjing Agricultural University IACUC, and the protocol was approved by the IACUC with the project number: 2011BAD34B02. According to the OIE Terrestrial Manual 2012 Chapter 2.3.14, the chickens that were alive but presented somnolence, apathy, akinesia or dyspnea were killed humanely by euthanasia of CO_2_ gas after the observation period, but the humane endpoints could not be used prior to the 14-day observation period in order to compare the therapeutic effect of five combinations scientifically objectively. And the conditions of chicken were monitored twice a day. During whole experimental session, the chickens were carefully nursed to reduce all kinds of stress, each process was strictly in accordance with the regulation of animal protection committee to minimize the injury.

#### Animals

One-day-old White Roman chickens (male) were purchased from Tangquan Poultry Farm and housed in wire cages (100 cm×60 cm×40 cm) in air-conditioned rooms at 37°C with 24 h light at the beginning of the pretrial period. The temperature was gradually decreased to room temperature and the light to 12 h per day. These parameters were maintained during the following days. The chickens were fed with commercial diet provided by the Feed Factory of Jiangsu Academy of Agricultural Science. All procedures involving animals were conducted in strict accordance with the Chinese legislation on the use and care of laboratory animals, and the experimental use of animals and procedures were approved by the Nanjing Agricultural University Animal Care Committee. During whole experiment, the chickens were carefully nursed to reduce all kinds of stress, each process was strictly in accordance with the regulation of animal protection committee to minimize the injury. All efforts were made to minimize suffering.

#### Experimental design

Two hundred and ten 28-day-old chickens with an average maternal antibody ND-HI titer of 3.17 Log_2_ were randomly assigned into 7 groups and challenged with 0.5 mL NDV at 10 LD_50_ by intramuscular injection, except the blank control (BC) group. After 24 h, the chickens in the five drug combinations groups were administered by drinking with SP_9_-sCP_1_, CP_7_-sCA_3_, EP_7_-sAP_3_, CA_9_-sEP_1_, and EP_7_-sCA_3_ at 8 mg·(㎏·W)^-1^, once a day for three successive days. The challenge control (CC) and BC groups were not treated. The nosogenic status and mortality of the chickens were observed daily. On day 14 after challenge, the morbidity, mortality and cure rate in each group were calculated according to the following formulas: morbidity (%) = the total number of chickens with clinical symptoms / the number of samples × 100%, mortality (%) = the total number of dead chickens / the number of samples × 100%, and cure rate (%) = the number of chickens without clinical symptoms on D_14_ / the number of samples × 100%. The humane endpoint was used at the end of survival study: the criteria for euthanasia using CO_2_ gas when animals displaying somnolence, apathy, akinesia or dyspnea on day 14 after challenge and the animal corpse for harmless treatment.

### Statistical analysis

The *A*_570_ value is expressed as the mean ± S.D, and Duncan’s multiple range test was used to analyze the difference among groups. The χ^2^-test was used to analyze the difference in the virus inhibitory rate, morbidity, mortality and cure rate with the software SPSS 20.0. The differences were considered significant at *P* < 0.05.

## Results

### The antiviral activity in vitro of PS_9_-sPS_1_ combinations

The *A*_*570*_ values of each group are listed in Table 1. The *A*_570_ values in all PS_9_-sPS_1_ combinations at all concentrations were significantly higher those in the corresponding virus control group (*P* <0.05).

**Table 1:** The *A*_570_ values of every PS_9_-sPS_1_ group.

Concentration（μg·mL^-1^）	CP_9_-sCA_1_	EP_9_-sAP_1_	EP_9_-sCA_1_	CA_9_-sEP_1_	SP_9_-sCP_1_
7.813	0.316±0.005^b^	0.346±0.004^b^	0.300±0.004^b^	0.391±0.009^b^	0.474±0.005^b^
3.906	0.297±0.010^c^	0.277±0.006^e^	0.262±0.012^d^ ^e^	0.315±0.007^c^	0.403±0.007^c^
1.953	0.307±0.006^b^ ^c^	0.276±0.006^e^	0.252±0.003^e^	0.276±0.010^d^	0.344±0.015^d^
0.977	0.273±0.009^d^	0.292±0.003^d^	0.266±0.009^c^ ^d^	0.295±0.036^c^ ^d^	0.313±0.006^e^
0.488	0.276±0.010^d^	0.310±0.004^c^	0.275±0.006^c^	0.301±0.021^c^	0.296±0.006^e^
VC	0.222±0.006^e^	0.222±0.006^f^	0.222±0.006^f^	0.222±0.006^e^	0.262±0.010^f^
CC	0.633±0.004^a^	0.633±0.004^a^	0.633±0.004^a^	0.633±0.004^a^	0.583±0.015^a^

^a-f^ Column date without the same superscripts differ significantly (*P* <0.05). Values are mean ± S.D, *n* = 4/group.

### The antiviral activity in vitro of PS_7_-sPS_3_ combinations

The *A*_570_ values of each group are listed in Table 2. Except for the SP_7_-sCP_3_ at 7.813 μg·mL^−1^ group, the *A*_*570*_ values in the other prescription groups at the other concentrations were significantly higher than those in corresponding virus control group (*P* < 0.05).

**Table 2:** The *A*_*570*_ values of every PS_7_-sPS_3_ group.

Concentration（μg·mL^-1^）	CP_7_-sCA_3_	EP_7_-sAP_3_	EP_7_-sCA_3_	CA_7_-sEP_3_	SP_7_-sCP_3_
7.813	0.312±0.009^e^	0.324±0.005^d^	0.375±0.005^b^	0.305±0.004^c^ ^d^	0.283±0.006^d^ ^e^
3.906	0.336±0.004^d^	0.357±0.005^c^	0.353±0.008^c^	0.310±0.005^c^ ^d^	0.317±0.006^c^
1.953	0.416±0.006^b^	0.327±0.003^d^	0.321±0.007^d^	0.325±0.002^b^	0.312±0.006^c^
0.977	0.312±0.003^e^	0.292±0.007^e^	0.311±0.006^d^	0.297±0.005^d^	0.299±0.006^c^ ^d^
0.488	0.379±0.002^c^	0.384±0.004^b^	0.308±0.007^d^	0.314±0.006^b^ ^c^	0.344±0.003^b^
VC	0.260±0.008^f^	0.260±0.008^f^	0.260±0.008^e^	0.260±0.008^e^	0.262±0.010^e^
CC	0.554±0.013^a^	0.554±0.013^a^	0.554±0.013^a^	0.554±0.013^a^	0.583±0.015^a^

^a-f^ Column date without the same superscripts differ significantly (*P* <0.05). Values are mean ± S.D, *n* = 4/group.

### The antiviral activity in vitro of PS_6_-sPS_4_ combinations

The *A*_570_ values of each group are listed in Table 3. The *A*_*570*_ values in CP_6_-sCA_4_, EP_6_-sAP_4_, and SP_6_-sCP_4_ at all concentrations were significantly higher than those in corresponding virus control group (*P*<0.05). The EP_6_-sCA_4_ at 7.813 μg·mL^−1^ group and CA_6_-sEP_4_ at 3.906 and 0.977 μg·mL^−1^ groups were significantly higher than those in corresponding virus control group (*P* <0.05).

**Table 3:** The *A*_570_ values of every PS_6_-sPS_4_ group.

Concentration（μg·mL^-1^）	CP_6_-sCA_4_	EP_6_-sAP_4_	EP_6_-sCA_4_	CA_6_-sEP_4_	SP_6_-sCP_4_
7.813	0.309±0.007^c^	0.323±0.006^b^	0.324±0.006^b^	0.266±0.004^c^	0.326±0.003^b^
3.906	0.306±0.005^c^	0.320±0.005^b^	0.274±0.009^c^	0.298±0.006^b^	0.310±0.007^b^
1.953	0.355±0.009^b^	0.315±0.012^b^	0.273±0.007^c^	0.274±0.014^c^	0.319±0.008^b^
0.977	0.299±0.007^c^	0.282±0.009^c^	0.272±0.013^c^	0.294±0.006^b^	0.327±0.002^b^
0.488	0.346±0.005^b^	0.328±0.006^b^	0.259±0.011^c^	0.243±0.015^d^	0.311±0.007^b^
VC	0.267±0.004^d^	0.267±0.004^d^	0.267±0.004^c^	0.267±0.004^c^	0.262±0.010^c^
CC	0.592±0.016^a^	0.592±0.016^a^	0.592±0.016^a^	0.592±0.016^a^	0.583±0.015^a^

^a-d^ Column date without the same superscripts differ significantly (*P* <0.05). Values are mean ± S.D, *n* = 4/group.

### The virus inhibitory rates of all combinations

**Fig 2:**
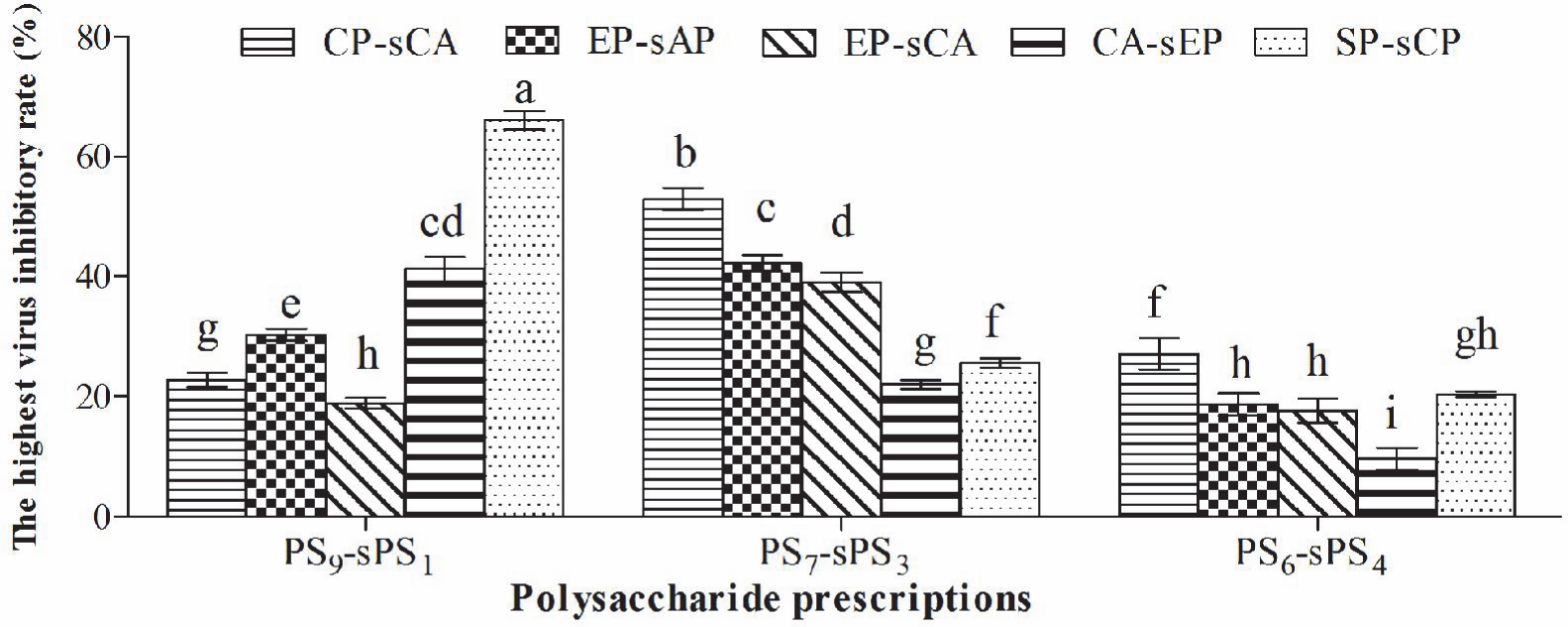
The highest virus inhibitory rates in all prescription groups. ^a-h^ Bars marked without the same letters differ significantly (*P* <0.05).

The highest virus inhibitory rates of all combinations are illustrated in Fig. 2. For the three ratios of each prescription, the highest virus inhibitory rates were observed in the CP_7_-sCA_3_, EP_7_-sAP_3_, EP_7_-sCA_3_ and SP_9_-sCP_1_ groups and were significantly higher than those in other two ratio groups (*P* <0.05). Among the fifteen combinations, the virus inhibitory rate in SP_9_-sCP_1_ group was the highest (66.12%) and significantly higher than those in other fourteen groups (*P* <0.05). The second highest was the CP_7_-sCA_3_ group (52.89%) and was significantly higher than the other thirteen groups (*P*<0.05), followed by the EP_7_-sAP_3_ (42.18%), CA_9_-sEP_1_ (41.20%) and EP_7_-sCA_3_ (39.0%) groups.

### Effects of SP_9_-sCP_1_ on NDV structure

**Fig 3:**
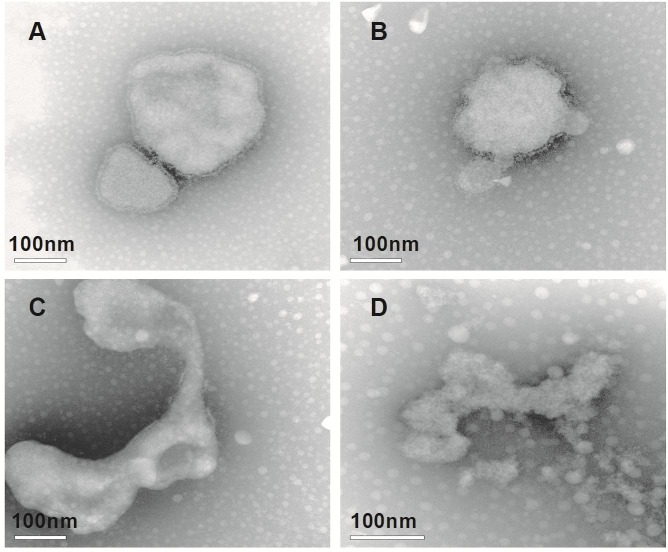
The changes of NDV structure in every group. A) Virus control group. B) Experimental group after incubation of 30 min. C) Experimental group after incubation of 60 min. D) Experimental group after incubation of 120 min.

The changes in the NDV structure for each group are illustrated in Fig. 3. For the control group, the NDV had a complete structure, with a round, fusiform or polymorphic shape, a compact envelope and a diameter of 100 nm to 300 nm (Fig. 3 A). For the experimental group, the structures of NDV were gradually destroyed with prolonged incubation time. At 30 min after incubation, the viral envelope was partially ruptured (Fig. 3 B). At 60 min, the viral cilia were not compact (Fig. 3 C), and at 120 min, the virus was destroyed into pieces (Fig. 3 D).

### Effect of SP_9_-sCP_1_ on NDV antigen expression

**Fig 4:**
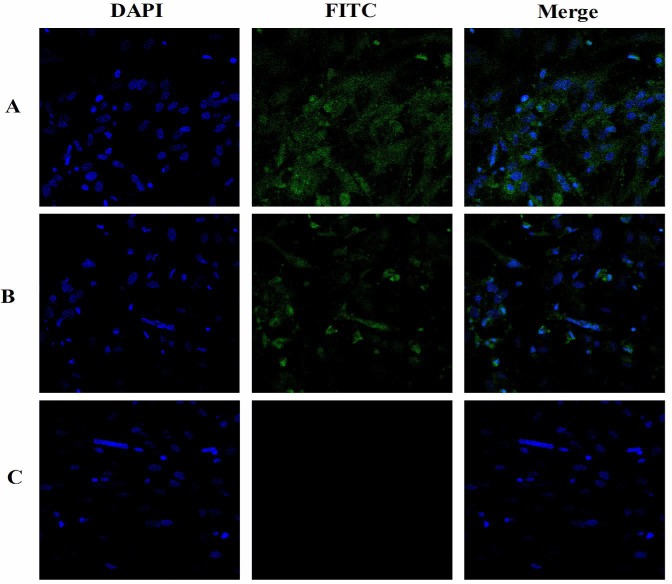
The changes of fluorescence in every group. A) Virus control group. B) Experimental group. C) Cell control group.

The changes in the fluorescence for each group are illustrated in Fig. 4. In the cell control group, almost no fluorescence intensity was presented (Fig. 4 C), the pixel intensity was 0.15 ±0.05 (pixel×10^2^/μm^2^). In the virus control group, the strongest fluorescence intensity was presented (Fig. 4 A), the pixel intensity was 22.52 ±8.14 (pixel×10^2^/μm^2^). In the experimental group, the fluorescence intensity was decreased (Fig. 4 B), the pixel intensity was 9.52 ±5.85 (pixel×10^2^/μm^2^) which was significantly weaker than that of virus control group (*P*<0.05).

### The curative effects in vivo

**Fig 5:**
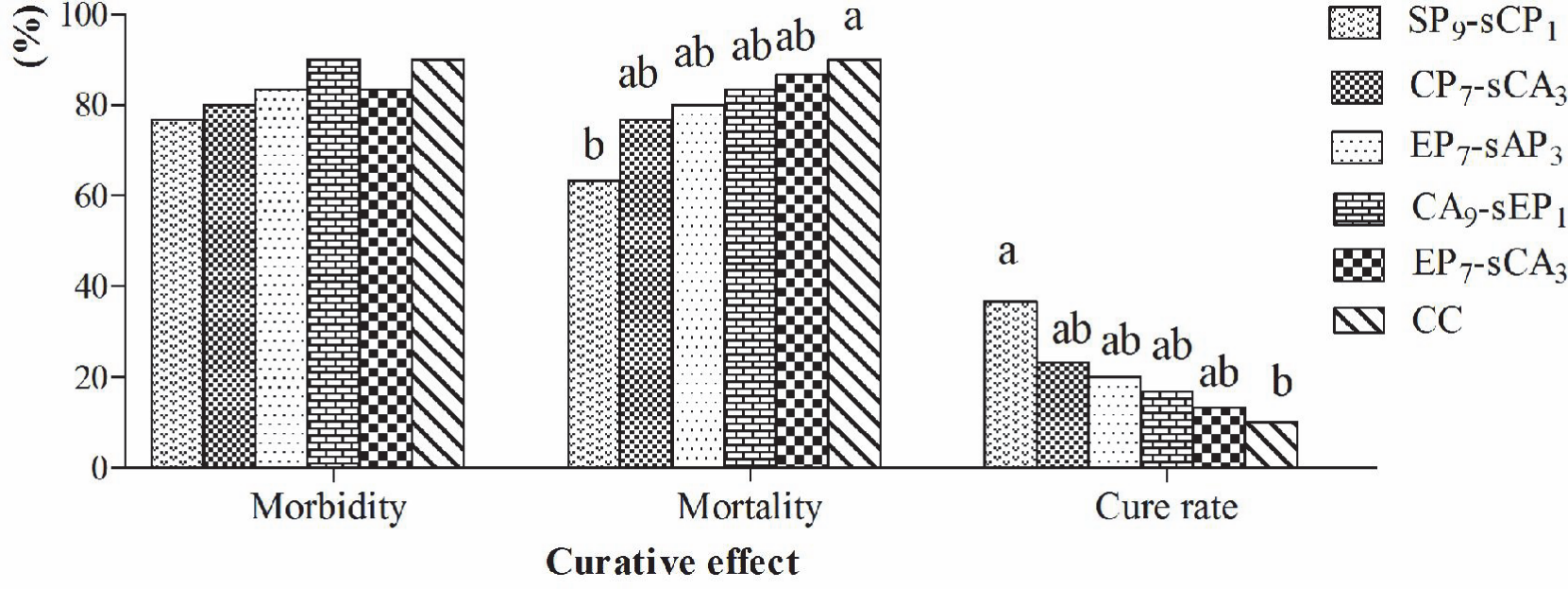
The morbidity, mortality and cure rate in every group. ^a-b^ Bars in the same index marked without the same letters differ significantly (*P* <0.05).

The results of this experiment are illustrated in Fig. 5. After challenge, the morbidities of all groups ranged from 76%-90% without significant differences (*P*>0.05). The mortality in the CC group was the highest, and the SP_9_-sCP_1_ group was the lowest (63.33%), which was significantly lower than that in CC group (*P*<0.05). The cure rates in the five drug combination groups were higher than the CC group. The SP_9_-sCP_1_ group was the highest and was significantly higher than the CC group (*P*<0.05).

## Discussion

In this study, the antiviral activities of five PS-sPS combinations were compared at three different proportions according to the carbohydrate content. The results of vitro experiments showed that at 9:1 ratio of PS-sPS, the cell *A*_570_ higher than the corresponding virus control group. At the 7:3 ratio, the *A*_570_ values in PS-sPS groups, except for the SP_7_-sCP_3_ at 7.813 μg·mL^−1^ group, were significantly higher than the corresponding virus control group. At the 6:4 ratio, the *A*_570_ values in the remaining PS-sPS groups, except for the EP_6_-sCA_4_ and CA_6_-sEP_4_ groups, were significantly higher than the corresponding virus control group. This indicated that these combinations at those ratios and concentrations possess significant antiviral activity.

The virus inhibitory rate directly reflects the antiviral potency [12]. The virus inhibitory rates of the three ratios of each combination were compared, CP-sCA, EP-sAP and EP-sCA at 7:3, and CA-sEP and SP-sCP at 9:1 presented the highest virus inhibitory rates, which were significantly higher than the other two ratios of the same prescription. This indicates that those ratios were the optimal for these combinations, which gives the combination the most potent antiviral activity. Of the 15 combinations, the virus inhibitory rate in the SP_9_-sCP_1_ group was the highest and was significantly higher than the other fourteen groups. This indicates that SP_9_-sCP_1_ possesses the highest antiviral activity.

To validate the antiviral activity of SP_9_-sCP_1_, its direct action on NDV was observed. The observation of virus structure by TEM showed that after the NDV was mixed with the SP_9_-sCP_1_, the NDV structures were gradually destroyed over time. At 30 min, the viral envelope was partially ruptured, at 60 min, the viral cilia were not compact, and at 120 min, the virus was destroyed into pieces. This indicates that SP_9_-sCP_1_ destroys the NDV structure. Immunofluorescence assays are widely used in antiviral research, and strong fluorescence indicates high antigen expression [20–22]. The results of the present study show that the fluorescence in the SP_9_-sCP_1_ treatment group was significantly reduced. This indicates that SP_9_-sCP_1_ inhibits NDV antigen expression. The viral life cycle includes attachment and entry into the host cell, transcription, replication and assemblage, these results confirmed that SP_9_-sCP_1_ could directly kill the virus before its attachment, or inhibit the transcription, replication and assemblage of the virus after its entry into host cell.

The efficacy of a drug must be clinically validated. Based on the in vitro results, the antiviral activities in vivo of five combinations with at the optimal ratio were compared using an NDV challenge test in chickens. The challenge was selected during the time when the chickens have lower maternal antibodies. The results of this study show that after challenge, the morbidities in all groups ranged from 76%-90% and were not significantly different. For the five groups, the mortalities were lower and the cure rates were higher than the CC group, particularly for the SP_9_-sCP_1_ group, which had the lowest mortality and the highest cure rate compared to the CC group. This demonstrates that the five combinations possess anti-NDV activity. The efficacy of the SP_9_-sCP_1_ was the highest, which is consistent with the results of the in vitro studies. Our previous studies showed that an epimedium polysaccharide and propolis flavone combination possessed improved anti-NDV activity [23].

SP_9_-sCP_1_ is a composite combination of SP and sCP. SP is a primary active ingredient of Solomonseal rhizonme, possess against herpes simplex virus in vitro [24], and CP is a primary active ingredient of *Codonopsis pilosula*. SP is composed of onefold fructose, relative molecular mass is 7073 [25]. Through further isolation two neutral polysaccharides, PSW-1a and PSW-1b-2 were obtained. PSW-1b-2 was characterized as a branched homogalactan, containing a 1,4-linked β-D-galactopyranosyl backbone with one β-D-galactopyranosyl stub substituted at O-6 of every 7th backbone residue, whereas PSW-1a as a highly branched galactomannan, possessing a 1,4-linked β-D-galactopyranosyl backbone, with a β-D-galactopyranosyl stub attached to O-6 of every 9th mannosyl residue [26]. CP is composed of β-D-galactopyranosyl, α-L-arabinose and α-D-rhamnopyranosyl, with a molecular mass of 1.1×10^4^ Da [27]. By isolation and purification with DEAE cellulose, five eluting peaks were obtained. Peak one made up the greatest proportion, it was a uniform polysaccharide with furan ring and high branched structure. Compositive monose included arabinose, ribose, mannose, fructose, galactose, glucose, and so on [28].

This study confirmed that SP and CP possessed antiviral activity, and the antiviral activity of CP was significantly enhanced by sulfation [29]. The activity of the sulfated polysaccharide was highly dependent on the DS [30, 31]. SO_4_^2-^ plays an important role in the antiviral activity of sulfated polysaccharides. It can combine with positive ions on the virus or cell surface to stereo-inhibit viral adsorption and prevent the virus from entering the cell or replicating in the cell. On the antiviral mechanism of the compounds may be that the compounds can inhibit the transcription, replication and assemblage of the virus invaded into host cell, thus relieve the destruction for the cells. This study confirmed that the antiviral activity of compound polysaccharides and sulfated polysaccharides changes with the ratio, and the antiviral activity of the drug combination is not higher with a larger proportion of sulfated polysaccharides. With an increase in the amount of sulfated polysaccharides, the antiviral activity is decreased. For example, SP_7_-sCP_3_, EP_6_-sCA_4_ and CA_6_-sEP_4_ had no antiviral activity_._ At a 9:1 ratio, the SP and sCP showed the optimal synergetic effect, and SP_9_-sCP_1_ possessed the strongest antiviral activity.

In conclusion, five drug combinations displayed antiviral activity in vitro and vivo. SP_9_-sCP_1_ exhibited the highest efficacy and may be a new type of antiviral drug.
